# Trends in Rates of ASIA Impairment Scale Conversion in Traumatic Complete Spinal Cord Injury

**DOI:** 10.1089/neur.2020.0038

**Published:** 2020-11-13

**Authors:** Ralph J. Marino, Michael Leff, Diana D. Cardenas, Jayne Donovan, David Chen, Steve Kirshblum, Benjamin E. Leiby

**Affiliations:** ^1^ Department of Rehabilitation Medicine, Philadelphia, Pennsylvania, USA.; ^7^ Biostatistics Division, Pharmacology and Experimental Therapeutics, Sidney Kimmel Medical College at Thomas Jefferson University, Philadelphia, Pennsylvania, USA.; ^2^ Sidney Kimmel Medical College at Thomas Jefferson University, Philadelphia, Pennsylvania, USA.; ^3^ Department of Physical Medicine and Rehabilitation, University of Miami Miller School of Medicine, Miami, Florida, USA.; ^4^ Kessler Institute for Rehabilitation, West Orange, New Jersey, USA.; ^5^ Department of Physical Medicine and Rehabilitation, Northwestern University Feinberg School of Medicine, Chicago, Illinois, USA.; ^6^ Rutgers New Jersey Medical School, Newark, New Jersey, USA.

**Keywords:** prognosis, recovery, traumatic spinal cord injury

## Abstract

Recent studies of persons with spinal cord injury (SCI) report higher conversion rates of the American Spinal Injury Association (ASIA) Impairment Scale (AIS) grades, especially for complete injuries. We examined the rate of conversion over time after complete SCI, accounting for demographic and injury characteristics. Subjects were 16 years of age and older with a complete SCI injury between 1995 and 2015, enrolled in the National SCI Database as day-1 admissions. We grouped subjects into 3-year intervals and assessed trends in conversion for the total sample and by tetraplegia (Tetra), high paraplegia (levels T1–9, HPara), and low paraplegia (levels T10–12, LPara).We used logistic regression to identify factors related to conversion such as age, sex, etiology, and level of injury. Of 2036 subjects, 1876 subjects had a follow-up examination between 30 and 730 days post-injury. Average age at injury was 34.2 ± 14.6 years; 79.8% were male, 44.6% Tetra, 35.3% HPara, and 20.1% LPara. There was a strong trend toward increased rates of conversion over time (*p* < 0.01 for all groups), especially for Tetra (to incomplete from 17.6% in 1995–1997 to 50% in 2013–2015, and to motor incomplete from 9.4% to 28.1%). Conversion rates for Para were less dramatic. There were increased odds of converting to incomplete for year of injury, level of injury (Tetra >LPara >HPara), non-violent etiology, and age (older is better). We found similar factors for conversion to motor incomplete, except sex was significant and etiology was not. Conversion rates from complete to incomplete and motor incomplete injury have been increasing, particularly for persons with tetraplegia. This has implications for acute clinical trials and for prognostication early after SCI.

## Introduction

Traumatic spinal cord injuries (SCI) immediately and drastically alter people's lives. Although many persons with SCI have some recovery, it remains difficult to predict who will recover due to the many heterogeneous characteristics of SCI.^1^

With the increasing number of therapies and treatment options under investigation for SCI, an accurate view of prognosis is important. Early-phase clinical trials, which focus on pharmacodynamics and safety, may use historical controls to evaluate a change in neurological status such as an American Spinal Injury Association (ASIA) Impairment Scale (AIS) grade conversion.^2,3^ The validity of this approach rests on the assumption that rates of conversion are stable over time, which may not be the case. Early studies on recovery after SCI indicated most persons (about 85%) with an AIS-A injury remained complete, with only a small percentage (6–8%) converting to motor incomplete.^4,5^ More recent studies have reported higher rates of conversion of AIS-A grade injuries.^6–8^ These studies have varied widely on inclusion criteria such as etiology of injury, level of injury, time period, and other factors that influence recovery rates. For example, Spiess and colleagues^6^ examined conversion rates for patients with SCI above the T10 neurological level enrolled in the European Multicenter Study about Spinal Cord Injury (EM-SCI) between 2003 and 2007 and found that 30% of subjects who were initially complete converted to incomplete status by 1 year, with 13% converting to motor incomplete. Marino and associates^7^ looked at persons with cervical SCI enrolled in the SCI Model Systems (SCIMS) database between 1994 and 2009 and found that 29.8% converted to incomplete and 15.1% to motor incomplete. Lee and co-workers^8^ examined recovery after thoracic-level SCI and reported that only 15.5% converted to incomplete, with 7.7% converting to motor incomplete. Roach and colleagues^9^ compared conversion rates in SCI due to blunt versus penetrating trauma, and showed that 24.1% of blunt versus 14.5% of penetrating injuries converted to incomplete, with 12.8% versus 8.7%, respectively, converting to motor incomplete.

Because variability in study populations can affect conversion rates, whether the apparent increase in rates of conversion for AIS-A injuries is real or due to population differences among the studies is not clear. The objective of this study was to examine rates of recovery over time in individuals with AIS-A SCI, accounting for level of injury, etiology, age, and sex.

## Methods

### Subjects

Data were obtained from the National Spinal Cord Injury Statistical Center (NSCISC), which houses the SCIMS database. This database contains the demographics, injury characteristics, functional outcomes, medical outcomes, and other information on persons with SCI enrolled in the longitudinal SCIMS study from 29 centers in the United States.

To be included in our study, individuals had to: 1) have been injured between 1995 and 2015, inclusive; 2) have been admitted to a participating center within 1 day of injury; 3) have been at least 16 years old at the time of injury; 4) have been examined for International Standards for Neurological Classification of SCI (ISNCSCI) classification within 14 days of injury; 5) have been classified as AIS grade A with a neurological level of injury at level T12 or above; and 6) have consented to participate in the longitudinal SCIMS study. To avoid atypical cases with extended motor function but no sacral sparing, we excluded subjects with injury above level T12 with a lower extremity motor score (LEMS) >0 or subjects with a T12 neurological level with motor function below L3 (>3 levels below motor level). This excluded 72 subjects from the analyses.

A total of 2036 subjects met the above criteria. There were 29 subjects reclassified from tetraplegia to paraplegia. These subjects had a sensory level below T1 and their upper extremity muscle scores were mostly grade 4 or 5. Note that before 2000 a muscle graded 4 could be considered normal strength if the examiner thought there were inhibiting factors.^10^ Of the total 2036 subjects, 1876 had data from a follow-up exam between 30 and 730 days from their initial injury. If more than one exam was available, we used the last exam performed.

The variables included in the SCIMS database have changed over time. Generally, the database is reviewed at the end of each funding cycle and variables are added or removed for the following funding cycle.^11^ Sensory and motor levels have been included in the database since 1993. The AIS grade was added in 1993, replacing the Frankel grade. Motor scores of individual key muscles were added in 1993. In 2006 the variables voluntary anal sphincter contraction (VAC) and any anal sensation (deep anal pressure, DAP) were added. Although individual dermatome light touch and pinprick scores were added in 2011, they were only included for admission to rehab, discharge, and 1-year follow-up. Dermatome sensory scores for day-1 admissions were not added to the database until 2016.

Severity of SCI was assessed using the AIS, which grades the severity of SCI using grades A–E.^12^ AIS grade A is “complete,” with no sensory or motor function in the sacral segments S4–5. AIS grades B–D are “incomplete.” AIS grade B denotes a “sensory incomplete” injury with the presence of sensory sacral sparing. AIS grade C is “motor incomplete,” where more than half of key muscles below the neurological level of injury have a muscle grade <3, whereas AIS grade D is “motor incomplete” with at least half of the key muscles below the neurological level of injury having a muscle grade ≥3. AIS grade E, “normal,” denotes SCI that has recovered to normal motor and sensory function.^12^ We define the term “conversion to incomplete” as a change from AIS-A to AIS grades B–E, and the term “conversion to motor incomplete” as a change from AIS-A to AIS grades C–E.

### Statistical analysis

Follow-up exams were checked for correct AIS grading using a classification algorithm developed by one of the authors, using the available neurological data. The algorithm accepted the designated sensory levels because there were no light touch or pinprick scores available to confirm the designation. The motor level was determined using the sensory level and the muscle grades according to the motor level definition in the 2011 standards.^13^ For examinations without DAP or VAC, the AIS grade was considered complete if designated grade A in the database, and incomplete if designated grades B–E. There were 60 cases where the AIS grade determined by the algorithm differed from the grade found in the database. The classification was confirmed by manual review of sensory and motor data.

The data were grouped into 3-year intervals, and trends in AIS grade conversion over time were assessed for the total sample, tetraplegia (Tetra), high paraplegia (T1–9, HPara) and low paraplegia (T10–12, LPara). Demographic data were compared using chi-square for categorical variables and *t* test for continuous variables. Trends over time for etiology of injury, level of injury, and time of initial examination were assessed using multi-nomial logistic regression and Spearman correlation coefficients.

Logistic regression was used to evaluate association of conversion to incomplete and to motor incomplete with year of injury (3-year groups), level of injury (Tetra vs. HPara and HPara vs. LPara), sex, etiology (violent vs. non-violent), race, and age (10-year groups). Univariable analyses were conducted first and then multi-variable analyses were performed to simultaneously model factors associated with conversion at the univariable level and factors associated with having a follow-up visit. Variables were checked for collinearity. Generalized estimating equation (GEE) methods with Wald *p*-values and confidence intervals were used to account for clustering by clinical center. All analyses were performed using SAS version 9.4 and SAS/STAT version 15.1 (SAS Institute, Cary, NC, USA).

## Results

### Sample characteristics

Characteristics of the sample and a comparison of those with and without follow-up exams are found in Table 1. The sample was predominantly male and over half were White. The most frequent causes of injury were vehicular, followed by violence and falls. Etiology was related to race and age at injury. Whites had the lowest rate of violent injury (6.9%), whereas Blacks had the highest (55.4%). Those with violent etiologies were most likely to have HPara level of injury (48.8%), whereas those with non-violent etiologies were most likely to have Tetra level of injury (51.2%). The most frequent cause of injury was motor vehicle crashes for subjects age 50 years or younger (43.2%), whereas falls were the most frequent etiology for subjects over age 50 (52.4%). Compared with those without follow-up data, those with follow-up data were younger (mean age 34.2 ± 14.6 vs. 44.2 ± 21.2 years, *p* < 0.001), were less likely to be Black, and were less likely to have falls or “other” etiology of injury.

**Table 1.** Demographics of Study Sample

	Total (*n*)	%	Followed (%)	No follow-up (%)	P-value
	2036		92.1	7.9	
Gender^a^					0.94
Male	1623	79.8	92.1	7.9	
Female	412	20.2	92.2	7.8	
Race^b^					0.006
White	1151	56.6	92.9	7.1	
Black	581	28.6	89.2	10.8	
Hispanic	245	12.1	94.3	5.7	
Other	55	2.7	98.0	2.0	
Etiology^b^					0.004
Vehicular	837	41.2	93.3	6.7	
Violence	498	24.5	92.2	7.8	
alls	456	22.4	89.5	10.5	
Sports	168	8.3	96.4	3.6	
Other	77	3.6	84.9	15.1	
Level of injury					0.99
Tetra (C1-8)	909	44.6	92.1	7.9	
HPara (T1-9)	718	35.3	92.2	7.8	
LPara (T10-12)	409	20.1	92.2	7.8	

^a^ One missing; ^b^four missing.

HPara, high paraplegia; LPara, low paraplegia; Tetra, tetraplegia.

The number of subjects per 3-year interval declined over time, from 398 in the first period to 186 in the final period. Time from injury to the initial examination increased over time, with the percentage of exams performed on the day of injury declining from 55.8% in the 1995–1997 year period to only 18.8% in the 2013–2015 year period. The median time to the follow-up exam dropped after the 1995–1997 year group (median 340 days, interquartile range [IQR] 97–462), and fluctuated over the remaining time periods from 117–262 days (see Supplementary Tables S1, S2, S3, S4). There was no difference in the trends in time to follow-up exams by level of injury (*p* = 0.56).

There were fluctuations but no clear trends in age at injury over time (see Supplementary Tables S1, S2, S3, S4). Subjects with tetraplegia were older than those with paraplegia (*p* < 0.005). The average age at injury of subjects increased over time for all three level of injury groups (*p* = 0.001), but there were no differences in trends in age across lesion groups (*p* = 0.95). Sex distribution did not change over time (*p* = 0.21) and did not differ by level of injury (*p* = 0.32).

### Overall conversion rates

**FIG. 1.**
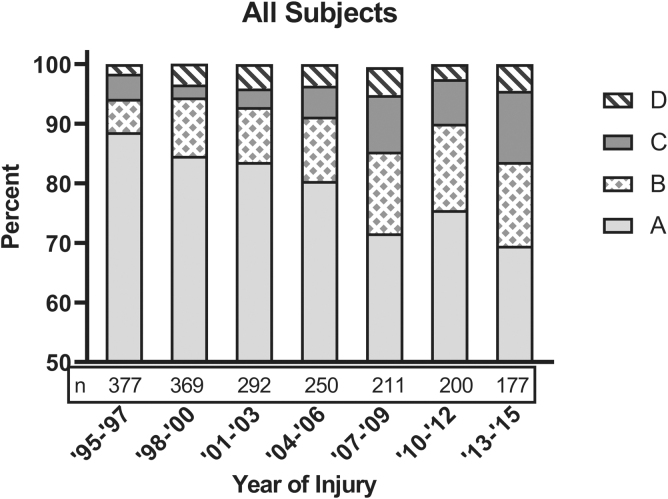
Trends in final American Spinal Injury Association (ASIA) Impairment Scale (AIS) grade for individuals with spinal cord injury initially classified as complete. Results represent unadjusted percentages.

**FIG. 2.**
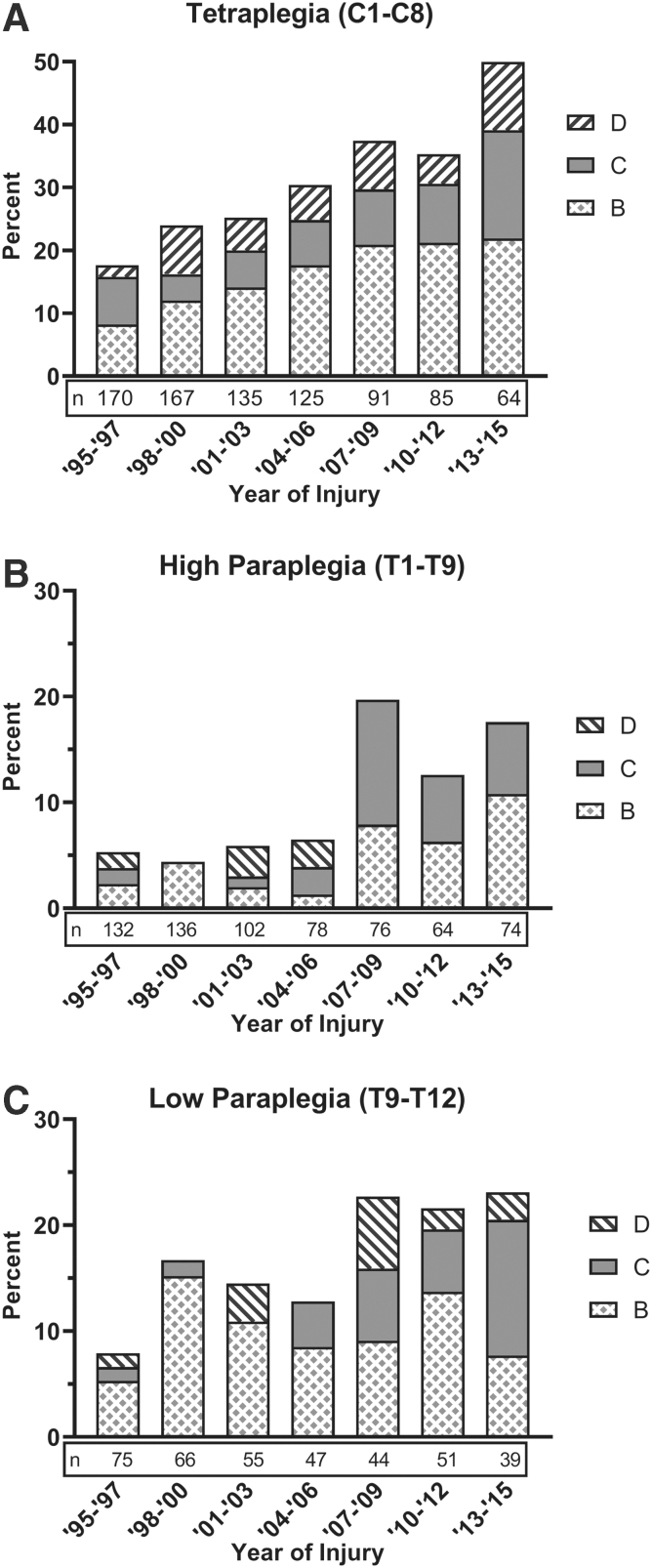
Trends in change in American Spinal Injury Association (ASIA) Impairment Scale (AIS) grade for individuals with spinal cord injury initially classified as complete. **(A)** Percentages of incomplete by AIS grade for tetraplegia (Tetra; C1–8). **(B)** Percentages of incomplete by AIS grade for high paraplegia (HPara; T1–9). **(C)** Percentages of incomplete by AIS grade for low paraplegia (LPara; T10–12).

For the entire sample the rate of conversion to incomplete was 19.2% and to motor incomplete was 8.8%. The rate of conversion increased over time (Fig. 1). In 1995–1997 compared with 2013–2015, conversion to incomplete increased from 11.4% to 30.5%, and conversion to motor incomplete increased from 5.8% to 16.4%. Individuals with cervical injuries had the largest increase of rate of conversion over the years, rising from 17.6% in 1995–1997 to 50.0% in 2013–2015 (Fig. 2A). Conversion rates for individuals with paraplegia improved, but not as dramatically as for tetraplegia. For HPara, rates of conversion to incomplete increased from 5.3% in 1995–1997 to 17.6% in 2013–2015 (Fig. 2B); for LPara the rates increased from 8.0% to 23.1% over the same period (Fig. 2C).

Most conversions to motor incomplete (83%) were based on motor function more than three levels below the motor level in participants with incomplete injuries; only 17% were based on VAC. Although VAC was not recorded in 82 participants classified as motor incomplete, all but 2 of these had motor function more than three levels below the motor level. By lesion level, 9% (10/113) of Tetra were classified as motor incomplete based on VAC alone, as were 30% (9/30) of HPara and 43% (10/23) of LPara.

### Predictors of conversion to incomplete

In univariable analyses (Table 2), conversion to incomplete was associated with year of injury, non-violent etiology, level of injury, and age (all *p* < 0.001), but not sex (*p* = 0.078) or race (0.077). Similar results were found in the multi-variable analysis, although the effect of age was somewhat reduced (odds ratio [OR] = 1.15 in univariable analysis vs. 1.08 [*p* = 0.017] in multi-variable analysis). Odds of converting to incomplete increased 1.23 times with every 3-year interval, and were 1.08 times greater per 10-year increase in age. Compared with HPara, LPara had a 1.98 times and Tetra a 3.81 times better odds of converting to incomplete. The odds of converting to incomplete decreased by 0.62 for persons with violent compared with non-violent etiology.

**Table 2.** Univariable and Multi-Variable Regression Analyses for Conversion from Complete to Incomplete

Variable	Comparison	Univariable**OR (95% CI)	P-value	Type 3*P*-value	Multi-Variable**OR (95% CI)	P-value	Type 3*P*-value
Level of injury	LPara vs. HPara	1.93 (1.21–3.08)	0.005	<0.0001	1.98 (1.19–3.28)	0.008	<0.0001
	Tetra vs. HPara	3.78 (2.92–4.88)	<0.0001		3.81 (2.94–4.92)	<0.0001	
Race	Black vs. White	0.77 (0.60–0.98)	0.036	0.077	0.96 (0.75–1.23)	0.754	0.613
	Hispanic vs. White	0.84 (0.61–1.14)	0.261		0.84 (0.60–1.19)	0.326	
	Other vs. White	1.04 (0.55–1.96)	0.905		1.01 (0.49–2.06)	0.982	
Sex	Female vs. Male	1.16 (0.98–1.38)	0.078		1.17 (0.99–1.39)	0.061	
Age	10-year increase	1.15 (1.08–1.22)	<0.0001		1.08 (1.01–1.14)	0.017	
Violent	Yes vs. No	0.46 (0.37–0.58)	<0.0001		0.62 (0.49–0.78)	<0.0001	
Year of injury	3-year increase	1.19 (1.12–1.28)	<0.0001		1.23 (1.14–1.33)	<0.0001	

CI, confidence interval; HPara, high paraplegia; LPara, low paraplegia; OR, odds ratio; Tetra, tetraplegia.

### Predictors of conversion to motor incomplete

In univariable analyses (Table 3), conversion to motor incomplete was associated with year of injury, level of injury, etiology, and age (for all *p* < 0.001), sex (*p* = 0.0164), and race (*p* = 0.0285). In multi-variable analyses, race and etiology were no longer significantly associated with conversion. Odds of converting to motor incomplete were 1.23 times greater for every 3-year interval, 1.38 times greater for females than males, and 1.15 times greater per 10-year increase in age. Tetra had over 3 times greater odds of converting compared with HPara. There was no significant difference in conversion to motor incomplete between HPara and LPara or between violent and non-violent etiologies.

**Table 3.** Univariable and Multi-Variable Regression Analyses for Conversion from Complete to Motor Incomplete

Variable	Comparison	Univariable**OR (95% CI)	P-value	Type 3*P*-value	Multi-Variable**OR (95% CI)	P-value	Type 3*P*-value
Level of injury	LPara vs. HPara	1.36 (0.68–2.73)	0.383	<0.0001	1.37 (0.66–2.87)	0.401	<0.0001
	Tetra vs. HPara	3.14 (2.09–4.73)	<0.0001		3.20 (2.14–4.78)	<0.0001	
Race	Black vs. White	0.73 (0.51–1.03)	0.074	0.028	0.89 (0.57–1.39)	0.604	0.238
	Hispanic vs. White	0.79 (0.51–1.23)	0.296		0.84 (0.57–1.24)	0.372	
	Other vs. White	0.35 (0.10–1.31)	0.119		0.31 (0.08–1.23)	0.096	
Sex	Female vs. Male	1.38 (1.06–1.80)	0.016		1.38 (1.07–1.78)	0.012	
Age	10-year increase	1.23 (1.10–1.36)	0.0002		1.15 (1.04–1.27)	0.005	
Violent	Yes vs. No	0.51 (0.36–0.73)	0.0003		0.84 (0.56–1.26)	0.403	
Year of injury	3-year increase	1.20 (1.10–1.30)	<0.0001		1.23 (1.12–1.35)	<0.0001	

CI, confidence interval; HPara, high paraplegia; LPara, low paraplegia; OR, odds ratio; Tetra, tetraplegia.

## Discussion

Overall, conversion of persons with SCI from complete to incomplete injury has increased in the span of 1995–2015, most notably for those with cervical SCI. The effect of advances in treatment on recovery after SCI remains unclear and is not well captured by the SCIMS database. There have been several changes in the management of traumatic SCI over the time period of this study. The American Association of Neurological Surgeons in 2002 recommended as an option maintaining mean arterial blood pressure (MAP) at 85 to 90 mm Hg during the first 7 days after acute SCI.^14^ There is some evidence that higher average MAP values are associated with improved neurological recovery, although avoiding hypotension may be the critical factor.^15^ Earlier decompression (within 24 h) for traumatic SCI has been associated with improved outcomes in both cervical and thoracolumbar injuries.^16–18^ Although the results for surgery sooner than 24 h post-injury have been mixed,^19,20^ a recent meta-analysis found better outcomes when surgery was performed within 12 h of injury.^16^

Persons with cervical injuries converted at a higher rate than those with thoracic-level injuries, which is consistent with current literature.^2^ However, the rate of conversion in the most recent group (2013–2015) was higher than other recent reports. Whether this represents a continued improvement in prognosis for cervical SCI or an unusually favorable sample remains to be seen.

For persons with paraplegia, those with LPara converted to incomplete almost twice as frequently as HPara, although there was no statistically significant difference between the groups when converting to motor incomplete. Similar differences in rates of conversion based on level of paraplegia have been found by others. Zariffa and colleagues^21^ found that the overall rate of conversion to incomplete for persons with complete thoracic paraplegia was 18.2%, increasing from 9.5% for those with an initial level from T2–5 to 29.2% for those with an initial level from T10–12. Persons with complete LPara also have a greater recovery of lower extremity motor points than those with higher thoracic injuries.^8,22^ This may be because the transition from spinal cord to cauda equina begins below the T10–11 disc level, with increasing numbers of nerve roots adjacent to the spinal cord progressing down to the tip of the conus.^23^ Recovery of strength in lower-extremity muscles could then in part occur via recovery of these nerve roots.

Unexpectedly, the odds of older persons converting to incomplete and motor incomplete were better than for younger persons by 8% and 15%, respectively. This was found in the univariable analysis and remained true after controlling for etiology of injury, level of injury, and sex. One possible explanation for this finding is that even within etiology groups, older individuals may have lower-energy injuries than younger individuals. Beck and associates^24^ found that SCI caused by low falls were more common in persons age 65 years or older, whereas high falls as a cause of SCI were more common in those younger than 65 years old. In addition, high falls were more likely to result in complete paraplegia compared with low falls (24% vs. 5.2%), which are less likely to convert to incomplete than tetraplegia.

Women were found to be 37% more likely to convert to motor incomplete compared with men, possibly due to sustaining lower-energy injuries. Women may have a better prognosis for recovery than men, presumably due to the neuroprotective effects of estrogen and progesterone.^25^ Female rats have been shown to have better functional recovery and greater tissue preservation at the injury epicenter than male rats in a thoracic contusion injury.^26^ Sipski and co-workers^27^ found that women with complete SCI had a greater improvement in total motor index score than men (*p* = 0.035) and a trend in AIS grade conversion (*p* < 0.1).

It is not uncommon in early-phase studies for researchers to use historical data of SCI conversion rates.^2^ Indeed, the International Campaign for Cures of Spinal Cord Injury Paralysis (ICCP) clinical guidelines panel published data in 2007 on spontaneous recovery after SCI to assist with trial planning.^28^ Although those data were useful at the time, the results of our study caution against continuing to use older historical data for current trial planning. The Phase 1/2a clinical trial of a rho antagonist in acute SCI found that 31% (5/16) of participants with cervical complete SCI converted to motor incomplete.^2^ Whereas this rate is double that reported by the ICCP panel,^28^ it is not much different from the 28% conversion rate we found for 2013–2015. If used, historical data should be as recent as possible, reflect the demographic and injury characteristics of the proposed study population, and account for changes in the standard of care. Registries involving many collection sites providing high-quality assessment data are preferred to single-center data. The number of known factors related to conversion (such as level of injury, age, and mechanism) and the uncertain effect of treatment interventions (such as pre-hospital management, type and timing of surgery, and blood pressure control) make the potential for confounding a concern even in a controlled clinical trial, where sufficient numbers of subjects are needed for randomization to be effective. Biomarkers derived from advanced magnetic resonance imaging techniques, neurophysiological assessments, and cerebral spinal fluid or serum may help to identify subjects with similar potential for improvement, but further research in this area is needed.^29–33^

Although the largest increase in conversions observed in this study were to sensory incomplete (AIS-B), it is promising to see conversions to motor incomplete nearly triple from 5.8% in 1995–1997 to 16.4% in 2013–2015. This change was even more pronounced in persons with tetraplegia, increasing from 9.4% to 28.1% in those time periods. When patients recover motor function, they may also regain some independence.^34^ However, one should not discount the importance of sensory sparing in persons with SCI. Compared with persons with AIS grade A injuries, persons with AIS grade B injuries at rehabilitation discharge are less likely to require indwelling catheters, report greater functional status, have fewer days hospitalized, and incur lower lifetime costs.^35,36^ The improved potential for recovery for persons with SCI classified as complete in the acute setting should be noted by clinicians in discussions with patients and family.

### Limitations

Although we have elucidated clear trends in conversion of SCI, we were not able to examine whether treatment factors impacted those trends because the NSCISC database does not include detailed information such as medical/surgical or rehabilitation treatments. Date of spine surgery is collected but not the precise time from injury to surgery, so that the effect of early surgery on conversion rates cannot be determined. The limited data on acute care interventions also leaves some findings unexplained, such as the more favorable rates of conversion in older compared with younger persons.

The number of subjects per 3-year interval declined over time. This was likely due in part to a decrease in the number of funded centers over time and a change in the specific centers in each funding cycle. From 1995–2000 there were 18 funded centers, which decreased to 16 centers from 2000–2006, and to 14 centers in 2006.^37,38^

We included patients who had a follow-up exam anywhere from 30 to 730 days post-injury. Longer follow-up times would be expected to result in greater rates of conversion.^1^ Our median times to follow-up tended to be shorter in the more recent time periods than the earlier time periods, which would tend to reduce the chances of detecting conversions in AIS grades. Therefore we do not think that length of follow-up biased our results.

Our ability to confirm the AIS grade classification in the database was limited by the lack of individual sensory dermatome scores in the database and sacral sparing information being included only recently. We were also unable to determine if there was a change in the extent of the zone of partial preservation over time that could contribute to the increased rates of conversion from complete to incomplete status. We excluded persons with a neurological injury level above T12 with any lower-extremity key muscle function, but were unable to do the same for sensory preservation. With the increasing age at injury, particularly in people with cervical level injuries, it is possible that the SCI resulted from lower-energy trauma with extended preservation of sensory function and a better chance for recovery. The importance of having complete ISNCSCI examination data in the database has been recognized. Since 2016 the neurological variables have included all dermatome and key muscle scores at all time-points where neurological examinations are performed: admission to system (day-1 admissions), admission to inpatient rehabilitation, discharge from rehabilitation, and 1-year post-injury.^11^

Although this study included a large number of participants, it is from a single registry where the contributing centers change every 5 years based on successful competition for grant awards. However, the database is unmatched in duration of enrollment and follow-up. The European Multicenter Study about SCI has been enrolling participants since about 2001 (Curt 2004)^39^ and the Rick Hansen SCI Registry since 2005.^40^ It would be valuable if researchers involved in these registries could assess trends in conversion in their databases to determine if they find similar increases in conversion rates over time and similar predictors of conversion.

## Conclusion

This study demonstrates increased rates of conversion of persons with SCI from complete to incomplete neurological status from 1995–2015 in the SCIMS database, especially for persons with tetraplegia. Confirmation of this trend in other SCI registries is encouraged. Clinicians in acute care settings should be aware of improved recovery rates for persons with SCI who initially have clinically complete injuries. Researchers involved in early-stage clinical trials in SCI should monitor trends in conversion rates so that studies are adequately powered. There is a need for biomarkers that can enhance the predictive value of the clinical exam to identify groups of patients with similar chances of recovery for clinical trials in acute SCI.

## Supplementary Material

Supplemental data

Supplemental data

Supplemental data

Supplemental data

## Abbreviations Used

AIS: American Spinal Injury Association Impairment Scale
ASIA: American Spinal Injury Association
CI: confidence interval
DAP: deep anal pressure
EM-SCI: European Multicenter Study about Spinal Cord Injury
GEE: Generalized estimating equation
HPara: high paraplegia
ICCP: International Campaign for Cures of Spinal Cord Injury Paralysis
IQR: interquartile range
ISNCSCI: International Standards for Neurological Classification of SCI
LEMS: lower extremity motor score
LPara: low paraplegia
MAP: mean arterial blood pressure
NSCISC: National Spinal Cord Injury Statistical Center
OR: odds ratio
SCI: spinal cord injury
SCIMS: Spinal Cord Injury Model Systems
Tetra: tetraplegia
VAC: voluntary anal sphincter contraction
