# Supplementary material for: Trends in Rates of ASIA Impairment Scale Conversion in Traumatic Complete Spinal Cord Injury
Source: Neurotrauma Rep. 2020 Nov 13;1(1):192–200. doi: 10.1089/neur.2020.0038 (PMC8240895; doi:10.1089/neur.2020.0038)
Supplement: Supplemental data [file Supp_TableS1.docx]

Supplemental Material:

**Article title:** Trends in rates of ASIA impairment scale conversion in traumatic complete spinal cord injury

Supplemental Table S1: Trends in age and sex by lesion level

| **Years** |  |  | **Age (years)** | | **Age > 50** | **Male** |
| --- | --- | --- | --- | --- | --- | --- |
| **Tetraplegia** | | **n** | **mean** | **sd** | **%** | **%** |
| '95-'97 |  | 170 | 34.5 | 14.7 | 14.1 | 78.8 |
| '98-'00 |  | 167 | 37.1 | 16.0 | 20.4 | 82.0 |
| '01-'03 |  | 135 | 34.5 | 15.0 | 15.6 | 82.2 |
| '04-'06 |  | 125 | 34.0 | 14.2 | 11.2 | 76.0 |
| '07-'09 |  | 91 | 39.4 | 18.0 | 25.3 | 75.8 |
| '10-'12 |  | 85 | 35.5 | 16.1 | 24.7 | 78.6 |
| '13-'15 |  | 64 | 41.1 | 17.1 | 34.4 | 81.2 |
| **High Paraplegia** | |  |  |  |  |  |
| '95-'97 |  | 132 | 30.8 | 14.3 | 9.8 | 81.1 |
| '98-'00 |  | 136 | 32.5 | 12.9 | 8.8 | 72.8 |
| '01-'03 |  | 102 | 31.9 | 12.3 | 12.8 | 78.4 |
| '04-'06 |  | 78 | 35.6 | 13.9 | 15.4 | 85.9 |
| '07-'09 |  | 76 | 33.3 | 14.4 | 15.8 | 82.9 |
| '10-'12 |  | 64 | 33.5 | 14.3 | 10.9 | 68.8 |
| '13-'15 |  | 74 | 34.6 | 13.1 | 21.6 | 83.8 |
| **Low Paraplegia** | |  |  |  |  |  |
| '95-'97 |  | 75 | 31.8 | 12.5 | 6.7 | 86.7 |
| '98-'00 |  | 66 | 29.4 | 11.4 | 4.6 | 80.3 |
| '01-'03 |  | 55 | 33.5 | 14.2 | 12.7 | 81.8 |
| '04-'06 |  | 47 | 31.4 | 11.1 | 4.3 | 78.7 |
| '07-'09 |  | 44 | 33.2 | 14.9 | 18.2 | 70.4 |
| '10-'12 |  | 51 | 33.5 | 12.2 | 11.8 | 82.4 |
| '13-'15 |  | 39 | 37.2 | 14.9 | 23.1 | 92.3 |
