# Supplementary material for: Trends in Rates of ASIA Impairment Scale Conversion in Traumatic Complete Spinal Cord Injury
Source: Neurotrauma Rep. 2020 Nov 13;1(1):192–200. doi: 10.1089/neur.2020.0038 (PMC8240895; doi:10.1089/neur.2020.0038)
Supplement: Supplemental data [file Supp_TableS2.docx]

Supplemental Table S2: Trends in days from injury to initial examination by lesion level

| **Years of Injury** | **N** | **Mean** | **sd** | **Median** | **IQR** | | |
| --- | --- | --- | --- | --- | --- | --- | --- |
| **Tetraplegia** |  |  |  |  |  |  |  |
| '95-'97 | 170 | 0.8 | 1.6 | 0 | 0 | - | 1 |
| '98-'00 | 167 | 1.2 | 1.7 | 1 | 0 | - | 2 |
| '01-'03 | 135 | 1.8 | 2.3 | 1 | 0 | - | 3 |
| '04-'06 | 125 | 2.0 | 2.8 | 1 | 0 | - | 3 |
| '07-'09 | 91 | 2.4 | 3.0 | 1 | 0 | - | 3 |
| '10-'12 | 85 | 2.6 | 3.2 | 2 | 0 | - | 4 |
| '13-'15 | 64 | 4.9 | 4.5 | 3 | 2 | - | 8 |
| **High Paraplegia** |  |  |  |  |  |  |  |
| '95-'97 | 132 | 1.0 | 1.9 | 0 | 0 | - | 1 |
| '98-'00 | 136 | 1.1 | 2.0 | 0 | 0 | - | 1 |
| '01-'03 | 102 | 1.6 | 2.3 | 1 | 0 | - | 2 |
| '04-'06 | 78 | 1.6 | 2.4 | 1 | 0 | - | 2 |
| '07-'09 | 76 | 2.0 | 2.4 | 1.5 | 0 | - | 3 |
| '10-'12 | 64 | 3.6 | 3.4 | 2 | 1 | - | 5.5 |
| '13-'15 | 74 | 4.0 | 3.6 | 3 | 1 | - | 7 |
| **Low Paraplegia** |  |  |  |  |  |  |  |
| '95-'97 | 75 | 1.4 | 2.6 | 0 | 0 | - | 2 |
| '98-'00 | 66 | 1.4 | 2.1 | 1 | 0 | - | 2 |
| '01-'03 | 55 | 1.3 | 1.4 | 1 | 0 | - | 2 |
| '04-'06 | 47 | 1.9 | 3.1 | 1 | 0 | - | 2 |
| '07-'09 | 44 | 1.4 | 1.3 | 1 | 0 | - | 2 |
| '10-'12 | 51 | 3.0 | 3.3 | 2 | 1 | - | 4 |
| '13-'15 | 39 | 3.5 | 3.0 | 3 | 1 | - | 5 |
