# Supplementary material for: Trends in Rates of ASIA Impairment Scale Conversion in Traumatic Complete Spinal Cord Injury
Source: Neurotrauma Rep. 2020 Nov 13;1(1):192–200. doi: 10.1089/neur.2020.0038 (PMC8240895; doi:10.1089/neur.2020.0038)
Supplement: Supplemental data [file Supp_TableS3.docx]

Supplemental Table S3: Trends in days from injury to final examination by lesion level

| **Year of Injury** | **N** | | | **Mean** | | **Std Dev** | | **Median** | | **IQR** | | | | | |
| --- | --- | --- | --- | --- | --- | --- | --- | --- | --- | --- | --- | --- | --- | --- | --- |
| **Tetraplegia** |  | | |  | |  | |  | |  | |  | |  | |
| '95-'97 | 170 | | | 310 | | 214 | | 304.5 | | 101 | | - | | 432 | |
| '98-'00 | 167 | | | 225 | | 179 | | 137 | | 86 | | - | | 347 | |
| '01-'03 | 135 | | | 255 | | 155 | | 282 | | 107 | | - | | 383 | |
| '04-'06 | 125 | | | 229 | | 155 | | 192 | | 81 | | - | | 358 | |
| '07-'09 | 91 | | | 196 | | 153 | | 131 | | 70 | | - | | 332 | |
| '10-'12 | 85 | | | 245 | | 197 | | 148 | | 74 | | - | | 378 | |
| '13-'15 | 64 | | | 193 | | 138 | | 137 | | 71 | | - | | 310 | |
| **High Paraplegia** |  | | |  | |  | |  | |  | |  | |  | |
| '95-'97 | 132 | | | 324 | | 220 | | 348 | | 95.5 | | - | | 498 | |
| '98-'00 | 136 | | | 243 | | 194 | | 156 | | 64.5 | | - | | 406.5 | |
| '01-'03 | 102 | | | 218 | | 164 | | 188.5 | | 59 | | - | | 371 | |
| '04-'06 | 78 | | | 228 | | 185 | | 140 | | 60 | | - | | 392 | |
| '07-'09 | 76 | | | 199 | | 161 | | 131.5 | | 61.5 | | - | | 341 | |
| '10-'12 | 64 | | | 183 | | 167 | | 83.5 | | 55 | | - | | 321 | |
| '13-'15 | 74 | | | 215 | | 168 | | 153 | | 59 | | - | | 391 | |
| **Low Paraplegia** | |  |  | |  | |  | |  | |  | |  | |  |
| '95-'97 | 75 | | | 328 | | 224 | | 363 | | 68 | | - | | 482 | |
| '98-'00 | 66 | | | 227 | | 182 | | 185 | | 55 | | - | | 379 | |
| '01-'03 | 55 | | | 243 | | 157 | | 300 | | 59 | | - | | 369 | |
| '04-'06 | 47 | | | 215 | | 164 | | 226 | | 50 | | - | | 375 | |
| '07-'09 | 44 | | | 261 | | 187 | | 262 | | 78.5 | | - | | 388.5 | |
| '10-'12 | 51 | | | 209 | | 187 | | 108 | | 52 | | - | | 353 | |
| '13-'15 | 39 | | | 229 | | 159 | | 284 | | 60 | | - | | 376 | |
