# Supplementary material for: Trends in Rates of ASIA Impairment Scale Conversion in Traumatic Complete Spinal Cord Injury
Source: Neurotrauma Rep. 2020 Nov 13;1(1):192–200. doi: 10.1089/neur.2020.0038 (PMC8240895; doi:10.1089/neur.2020.0038)
Supplement: Supplemental data [file Supp_TableS4.docx]

Supplemental Table S4: Trends in etiology by year of injury and age

| **Age 50 or younger** |  | **Etiology (%)** | | | | |
| --- | --- | --- | --- | --- | --- | --- |
| **Year of Injury** | N | **Vehicular** | **violence** | **sports** | **falls** | **other** |
| '95-'97 | 335 | 36.1 | 38.5 | 7.8 | 14.3 | 3.3 |
|  |  |  |  |  |  |  |
| '98-'00 | 320 | 39.4 | 25.6 | 11.9 | 21.6 | 1.6 |
|  |  |  |  |  |  |  |
| '01-'03 | 250 | 49.6 | 22.4 | 12.0 | 14.8 | 1.2 |
|  |  |  |  |  |  |  |
| '04-'06 | 222 | 51.4 | 18.5 | 9.5 | 17.6 | 3.2 |
|  |  |  |  |  |  |  |
| '07-'09 | 167 | 41.9 | 32.3 | 9.6 | 15.6 | 0.6 |
|  |  |  |  |  |  |  |
| '10-'12 | 166 | 44.0 | 29.5 | 7.2 | 18.1 | 1.2 |
|  |  |  |  |  |  |  |
| '13-'15 | 129 | 45.7 | 28.7 | 8.5 | 14.0 | 3.1 |
|  |  |  |  |  |  |  |
| **Age greater than 50** | |  |  |  |  |  |
| '95-'97 | 42 | 33.3 | 7.1 | 0.0 | 52.4 | 7.1 |
|  |  |  |  |  |  |  |
| '98-'00 | 49 | 36.7 | 0.0 | 6.1 | 51.0 | 6.1 |
|  |  |  |  |  |  |  |
| '01-'03 | 41 | 43.9 | 2.4 | 0.0 | 43.9 | 9.8 |
|  |  |  |  |  |  |  |
| '04-'06 | 28 | 35.7 | 3.6 | 0.0 | 42.9 | 17.9 |
|  |  |  |  |  |  |  |
| '07-'09 | 43 | 27.9 | 2.3 | 2.3 | 53.5 | 14.0 |
|  |  |  |  |  |  |  |
| '10-'12 | 33 | 27.3 | 0.0 | 6.1 | 60.6 | 6.1 |
|  |  |  |  |  |  |  |
| '13-'15 | 47 | 27.7 | 10.6 | 4.3 | 44.7 | 12.8 |
